# Rhesus Macaques (*Macaca mulatta*) Are Natural Hosts of Specific *Staphylococcus aureus* Lineages

**DOI:** 10.1371/journal.pone.0026170

**Published:** 2011-10-20

**Authors:** Sanne van den Berg, Willem J. B. van Wamel, Susan V. Snijders, Boudewijn Ouwerling, Corné P. de Vogel, Hélène A. Boelens, Rob J. L. Willems, Xander W. Huijsdens, Frank A. W. Verreck, Ivanela Kondova, Peter J. Heidt, Henri A. Verbrugh, Alex van Belkum

**Affiliations:** 1 Department of Medical Microbiology and Infectious Diseases, Erasmus MC, Rotterdam, The Netherlands; 2 Animal Science Department, Biomedical Primate Research Centre, Rijswijk, The Netherlands; 3 Department of Medical Microbiology, University Medical Centre Utrecht, Utrecht, The Netherlands; 4 Laboratory for Infectious Diseases and Screening, National Institute for Public Health and the Environment, Bilthoven, The Netherlands; 5 Department of Parasitology, Biomedical Primate Research Centre, Rijswijk, The Netherlands; Duke University Medical Center, United States of America

## Abstract

Currently, there is no animal model known that mimics natural nasal colonization by *Staphylococcus aureus* in humans. We investigated whether rhesus macaques are natural nasal carriers of *S. aureus*. Nasal swabs were taken from 731 macaques. *S. aureus* isolates were typed by pulsed-field gel electrophoresis (PFGE), *spa* repeat sequencing and multi-locus sequence typing (MLST), and compared with human strains. Furthermore, the isolates were characterized by several PCRs. Thirty-nine percent of 731 macaques were positive for *S. aureus*. In general, the macaque *S. aureus* isolates differed from human strains as they formed separate PFGE clusters, 50% of the isolates were untypeable by *agr* genotyping, 17 new *spa* types were identified, which all belonged to new sequence types (STs). Furthermore, 66% of macaque isolates were negative for all superantigen genes. To determine *S. aureus* nasal colonization, three nasal swabs from 48 duo-housed macaques were taken during a 5 month period. In addition, sera were analyzed for immunoglobulin G and A levels directed against 40 staphylococcal proteins using a bead-based flow cytometry technique. Nineteen percent of the animals were negative for *S. aureus*, and 17% were three times positive. *S. aureus* strains were easily exchanged between macaques. The antibody response was less pronounced in macaques compared to humans, and nasal carrier status was not associated with differences in serum anti-staphylococcal antibody levels. In conclusion, rhesus macaques are natural hosts of *S. aureus*, carrying host-specific lineages. Our data indicate that rhesus macaques are useful as an autologous model for studying *S. aureus* nasal colonization and infection prevention.

## Introduction

In the light of the rapid, worldwide emergence of antibiotic resistance in and the lack of an effective long-term elimination strategy against *Staphylococcus aureus* (*S. aureus*) nasal carriage, new approaches are needed to prevent staphylococcal carriage and its consequent diseases. Many different *S. aureus* animal models have been described for studying the pathogenesis of *S. aureus* colonization and infection. These models have provided insight into the role of bacterial virulence genes and have assisted in the estimation of vaccine efficacy. Models have been set up in various species, such as insects, worms, mice, rats, guinea pigs, hamsters, chickens, rabbits, sheep, dogs, pigs, and cows [1]–[4]. Notably, most of these animals, unlike humans, are not natural nasal carriers of *S. aureus*, only pigs, sheep, and cows may be naturally colonized by *S. aureus*. For instance, *S. aureus* sequence type (ST) 398 strains belong to a biotype associated with pigs and other species of livestock [5], [6]. *S. aureus* strain RF122 is a member of a bovine mastitis-associated clone that is genetically different from human clones of *S. aureus* [7], [8]. Humans can acquire these *S. aureus* strains during intensive short-term exposure to livestock, but in most cases the strain is lost again within 24 hours [9]. However, lack of a natural, human-like animal model of nasal *S. aureus* carriage is still a problem. Therefore, we investigated whether a non-human primate could provide a natural model for human nasal carriage of *S. aureus*.

The rhesus macaque (*Macaca mulatta*) belongs to the old world monkeys and has been used in several studies involving *S. aureus*. Kuklin *et al.* used rhesus macaques to study the immunogenicity of IsdB [10]. Protection against lethal SEB aerosol exposure by passive transfer of SEB-specific antibodies was also studied in macaques [11]. In addition to these protection studies, rhesus macaques were also used for safety evaluations. For example, the tolerability and potential toxicity of the thrombolytic agent staphylokinase was investigated in healthy rhesus macaques [12]. To our knowledge, natural nasal *S. aureus* carriage and the consequences on natural immunity in rhesus macaques has never been studied before.

Using a cross sectional setup, we isolated 287 *S. aureus* strains from 731 rhesus macaques after nasal sampling. We compared *S. aureus* strains isolated from rhesus macaques and humans. Furthermore, we followed a group of 48 rhesus macaques in time for studying persistence of nasal carriage of *S. aureus*. In addition, serum samples from these macaques were analyzed for anti-staphylococcal immunoglobulin G and A (IgG and IgA) levels.

## Materials and Methods

### Study population

For comparison of *S. aureus* strains isolated from rhesus macaques with those from humans, 731 rhesus macaques from the breeding colony of the Biomedical Primate Research Centre (Rijswijk, The Netherlands) were studied. These animals were of Indian, Burmese and Chinese origin. These macaques were housed in groups of 2–44 individuals. Furthermore, 48 young rhesus macaques that were recently imported from China were followed in time for studying the persistence of *S. aureus* nasal carriage as well as their serum anti-staphylococcal antibody levels. These animals were duo-housed in 4 different animal rooms. Physical contact with the macaques in the neighbouring cage was possible. In each room 2 groups of cages were located opposite to each other.

### Human *S. aureus* strains

For reasons of comparison, 56 human isolates of *S. aureus* were included. These carriage (n = 30) and bacteremia derived (n = 20) MSSA isolates have been described before [13], [14]. Three MSSA isolates from animal care-takers and 3 *S. aureus* strains for which the genome sequence is known were included as well (N315, Mu50, MRSA252).

### Ethics statement

Sampling of the longitudinally screened macaques was approved by the Animal Experiments committee of the Biomedical Primate Research Centre (Dierexperimentencommissie (DEC), which is the ethical committee installed and officially recognised as required by the Dutch Law on Experimental Animals and which is the Dutch analogue for the IACUC). The approval number is: DEC#579, dated October 28, 2008. The study was conducted in compliance with all relevant Dutch laws and in agreement with international and scientific standards and guidelines.

Sampling for bacteriology of the animals in the breeding groups was performed as part of their yearly routine health screening, which is by Dutch law not considered to be an animal experiment. Therefore no permission of the ethical committee was necessary for this part of the study. All samplings were performed under ketamine anaesthesia (which is routine for all health checks of non-human primates) and all efforts were made to minimize stress and suffering of the animals. The housing, care and handling of all animals were according to the Dutch Law on Experimental Animals and the European Directive 86/609/EEC. The research described is in accordance to the Weatherall Report recommendations for good welfare.

Human samples were described before [13], [14]. For carriage isolates and serum samples, volunteers provided written informed consent and the local Medical Ethics Committee of the Erasmus Medical Centre Rotterdam approved the study (MEC-2007-106). For bacteremia derived isolates, *S. aureus* strains were collected for routine culture. The Medical Ethics Committee of the Erasmus Medical Centre Rotterdam approved the study (MEC-2007-106, addendum 2).

### Sampling procedures

A total of 731 macaques were sampled once for nasal carriage of *S. aureus*, while another 48 macaques were screened three times during a 5 month period. Nasal cultures were taken by streaking both anterior nares using a sterile cotton swab (Swab Transystem, Greiner Bio One, Alphen aan de Rijn, The Netherlands) during regular animal medical check-up. All swabs were processed within 24 hours. Nasal swabs were plated on a Columbia sheep blood agar plate-medium (bioTRADING, Mijdrecht, The Netherlands). Plates were read after one and two days of incubation at 35°C. Identification of *S. aureus* was based on colony morphology and coagulase plasma test (Becton Dickinson, Breda, The Netherlands) and confirmed by API Staph (bioMérieux, Boxtel, The Netherlands).

### Pulsed-field gel electrophoresis

Pulsed-field gel electrophoresis (PFGE) of *Sma*I digested chromosomal DNA from all *S. aureus* strains from rhesus macaques and 56 strains from humans was performed as described previously [15]. Relatedness among the PFGE profiles was evaluated with Bionumerics software (version 3.0; Applied Maths, Ghent, Belgium). A dendrogram was produced using the Dice coefficient and an unweighted-pair group method using arithmetic averages (UPGMA). Band tolerance was set at 2.0%.

### Anti-staphylococcal antibodies

IgG and IgA antibody levels in serum directed against the following antigens were semi-quantified: *S. aureus* proteins clumping factor A and B (ClfA and ClfB); surface protein G (SasG); iron-responsive surface determinants A and H (IsdA and IsdH); fibronectin-binding proteins A and B (FnbpA and FnbpB); serine-aspartate dipeptide repeat protein D and E (SdrD and SdrE); staphylococcal enterotoxins A-E, G-J, M-O, Q, and R (SEA - SEE, SEG - SEJ, SEM - SEO, SEQ, SER); toxic shock syndrome toxin 1 (TSST-1); chemotaxis inhibitory protein of *S. aureus* (CHIPS); staphylococcal complement inhibitor (SCIN); extracellular fibrinogen-binding protein (Efb); exfoliative toxin A and B (ETA and ETB); alpha toxin; γ hemolysin B (HlgB); leukocidin (Luk) S-PV, LukF-PV, LukD-PV, and LukE-PV; and staphylococcal superantigen-like proteins 1, 3, 5, 9, and 11 (SSL1, SSL3, SSL5, SSL9, and SSL11). Antibodies were semi-quantified simultaneously in a single multiplex assay using a bead-based flow cytometry technique (xMap; Luminex Corporation). Methods have been described elsewhere [14], [16], [17], with the exception that a 1∶50 dilution of R-phycoerythrin (RPE)-conjugated AffiniPure goat anti-human secundairy IgA was used. Tests were performed in independent duplicates, and the median fluorescence intensity (MFI) values, reflecting semiquantitative antibody levels, were averaged. In each experiment, control beads (no protein coupled) were included to determine nonspecific binding. In the event of nonspecific binding, the nonspecific MFI values were subtracted from the antigen-specific results.

Serum samples from 47 out of the 48 rhesus macaques from China were analyzed. Anti-staphylococcal antibody levels in these sera were compared to those in sera from 20 Dutch healthy human volunteers [14]. Pooled serum from all rhesus macaques included in the present study or from humans was used as a standard.

### DNA isolation

DNA isolation of *S. aureus* was performed for representative isolates from each PFGE cluster. Total DNA of *S. aureus* was isolated with the QIAamp DNA Mini Kit (QIAGEN, Venlo, The Netherlands) according to the manufacturer's instructions, or with MagNA Pure LC DNA isolation kit III (bacteria, fungi) using the MagNA Pure LC instrument (Roche Diagnostics, Almere, The Netherlands) [18].

### *spa* genotyping

PCR for amplification of the *S. aureus* protein A (*spa*) repeat region was performed according to the published protocol [19]. PCR products were purified with the QIAquick PCR Purification Kit (QIAGEN, Venlo, The Netherlands) and sequenced using two amplification primers from a commercial supplier (SeqLab, Goettingen, Germany). The forward and reverse sequence chromatograms were analyzed with the Ridom StaphType software (Ridom GmbH, Würzburg, Germany).

### Multi-locus sequence typing

For one representative *S. aureus* isolate of each *spa* type, the sequence type (ST) was determined using multi-locus sequence typing (MLST) [20]. goeBURST [21], as implement in Phyloviz software (http://www.phyloviz.net/wiki/), and which used the same priority rules for linking STs as eBURST but with a global optimization, was used to infer relatedness between STs. goeBURST was run on the whole *S. aureus* MLST database (http://saureus.mlst.net/; interrogated July 2011) supplemented with STs of rhesus macaque isolates not present in the *S. aureus* MLST database at the time of interrogation. Clonal complexes (CCs) are defined as STs that are linked through single locus variants (SLVs) and are named on the basis of the predicted founder ST, which is the ST having the most SLVs.

### Staphylococcal antigen PCRs

Methicillin resistance was detected with *mecA*-specific primers [22]. Six sets of multiplex PCRs were established to amplify 19 superantigen genes (*sea* to *see*, *seg* to *ser*, *seu*, *tst*), *eta*, *etd* and *agr-1* to *-4*, as described previously [23]. The presence of the *scn*, *chp*, *clfA*, *clfB*, *efb*, *fnbA*, *fnbB* and *tst* genes was determined by PCR as well [13], [24]–[26].

### Statistical analysis

Statistical analyses were performed with SPSS software, version 15.0 (SPSS). The Mann-Whitney *U* test was used to compare median differences in anti-staphylococcal antibody levels. Differences were considered statistically significant when 2-sided *P*-values were <0.05.

## Results

### Characterization of *S. aureus* isolates in rhesus macaques

A total of 731 macaques was screened once for presence of *S. aureus* in their nasal cavity. Thirty-nine percent (287/731) of these cultures were positive for *S. aureus*, clearly indicating that rhesus macaques can be *S. aureus* nasal carriers.

**Figure 1:**
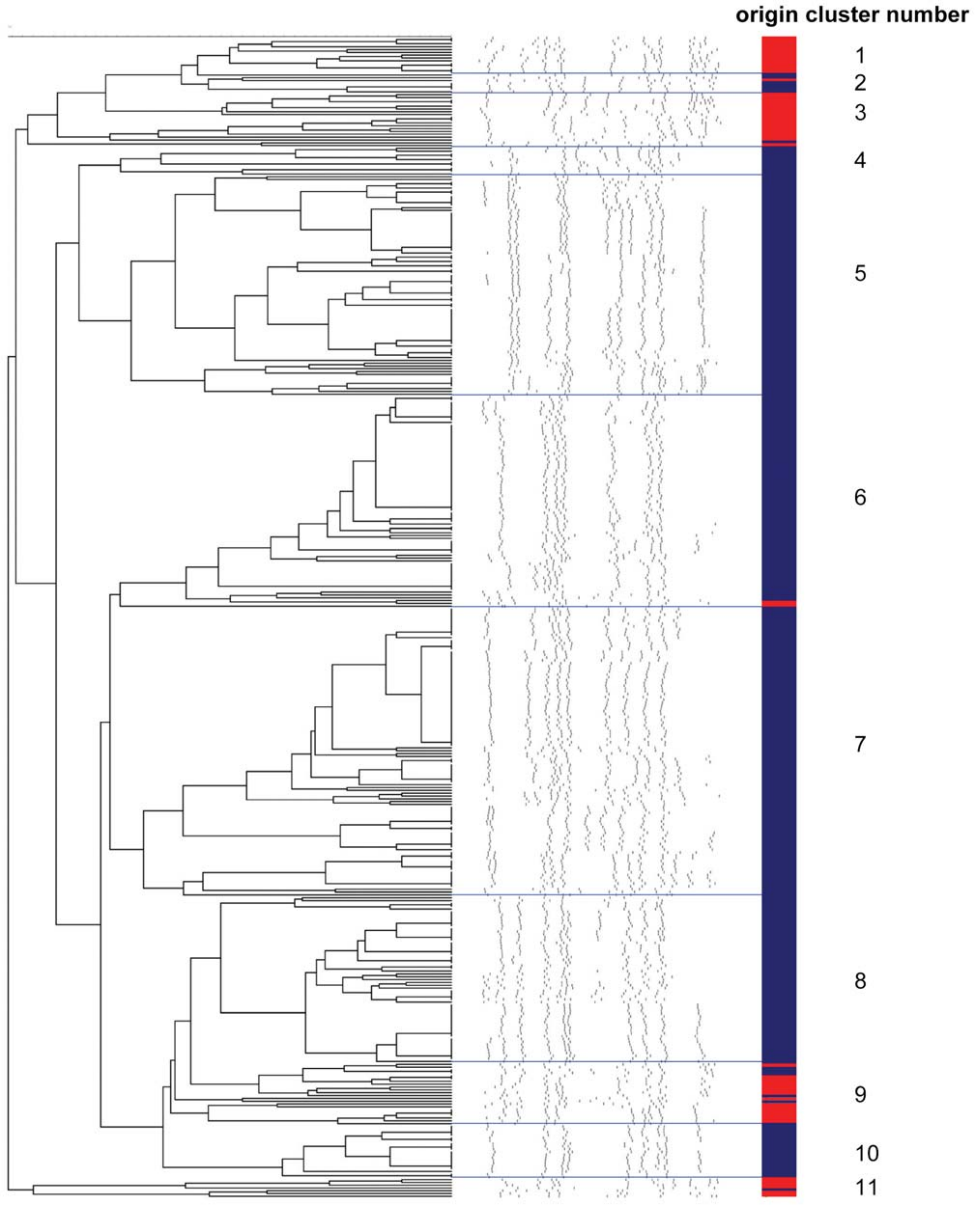
Dendrogram of the PFGE data of *S. aureus* strains isolated from rhesus macaques and humans. Blue color indicates *S. aureus* strains isolated from rhesus macaques, red color indicates *S. aureus* strains isolated from humans. Five clusters consisted of only rhesus macaque isolates, one comprised only human isolates, and five clusters included isolates from both macaques and humans.

*S. aureus* isolates from the 287 culture positive macaques as well as all collected isolates from the 48 macaques that were sampled three times (a total of 355 isolates) were typed using molecular methods including PFGE. This revealed 11 major clusters (Figure 1). Five of these consisted of only rhesus macaque isolates, one comprised only human isolates, while five clusters included isolates from both macaques and humans.

For further characterization of the *S. aureus* isolates from rhesus macaques, *spa* genotyping was performed on representative isolates from each PFGE cluster (n = 108) (Table 1). These isolates were all *spa*-positive, re-confirming that these were indeed *S. aureus* strains. This revealed 22 different *spa* types, of which five have already been described before. Consequently, we identified 17 new *spa* types not included in the Ridom SpaServer database at the time of analysis. These new *spa* types comprised 59% of the isolates. The most prominent clone was t516, which comprised 21 isolates (19%). *Spa* types t4168, t729 and t4167 were represented by 18 (17%), 15 (14%) and 11 (10%) isolates, respectively. Nine *spa* types were present as single isolates. Each major PFGE cluster comprised 1 to 8 different *spa* types, though the repeat succession of different *spa* types within a cluster were clearly related in some cases (Table 2).

**Table 1:** Different *spa* types found in 108 representative *S. aureus* strains isolated from rhesus macaques.

*spa* type		No. of isolates	Percentage of isolates
t189		1	0.9
t516		21	19.4
t729		15	13.9
t786		4	3.7
t1814		3	2.8
t4167	new	11	10.2
t4168	new	18	16.7
t4980	new	2	1.9
t4981	new	1	0.9
t5010	new	2	1.9
t5011	new	1	0.9
t5012	new	1	0.9
t5013	new	6	5.6
t5014	new	1	0.9
t5015	new	4	3.7
t5016	new	1	0.9
t5017	new	5	4.6
t5018	new	5	4.6
t5135	new	3	2.8
t5136	new	1	0.9
t5137	new	1	0.9
t5458	new	1	0.9

**Table 2:** Relatedness of *spa* types within each PFGE cluster.

PFGE cluster	*spa* types in PFGE cluster	Repeat succession																				
1	*-*																					
2	t4980	4	-	16	-	21	-	12	-	12	-	17	-	13	-	39	-	13				
	t5135	210	-	23	-	34	-	34	-	16	-	17	-	34	-	17	-	17	-	23	-	17
3	-																					
4	t516	8	-	16	-	2	-	25	-	51	-	68	-	2	-	24	-	2	-	24		
	t5014	15	-	12	-	16	-	16	-	2	-	25	-	34	-	24	-	17	-	24	-	17
	t5015	15	-	12	-	16	-	16	-	25	-	25	-	34	-	24	-	17	-	17	-	17
	t5017	8	-	34	-	2	-	43	-	34	-	43	-	43	-	16	-	2	-	17	-	83
5	t516			8	-	16	-	2	-	25	-	51	-	68	-	2	-	24	-	2	-	24
	t5011			8	-	16	-	16	-	25	-	25	-	34	-	24	-	17	-	17	-	17
	t5015	15	-	12	-	16	-	16	-	25	-	25	-	34	-	24	-	17	-	17	-	17
6	t516	8	-	16	-	2	-	25	-	51	-	68	-	2	-	24	-	2	-	24		
	t729	7	-	12	-	21	-	17	-	13	-	34	-	34	-	34	-	33	-	34		
	t786	7	-	12	-	21	-	17	-	13	-	34	-	34	-			33	-	34		
	t1814	7	-	12	-	21	-	17	-	34	-	34	-	34	-			33	-	34		
	t5012	8	-	34	-	2	-	43	-	34	-	43	-	43	-	16	-	2	-	17	-	16
	t5136	4	-	2	-	17	-	17	-	34	-	24	-	17	-	17	-	17				
	t5458	210	-	23	-	34	-	17	-	34	-	22	-	34	-	17	-	23	-	34		
7	t4167	7	-	23	-	17	-	22	-	249	-	12	-	117	-	24	-	25	-	17		
	t4168	8	-	23	-	21	-	17	-	13	-	13	-	13	-	24	-	23				
	t4980	4	-	16	-	21	-	12	-	12	-	17	-	13	-	39	-	13				
	t5013	7	-	23	-	23	-	17	-	34	-	21	-	12	-	20	-	24				
	t5016	8	-	34	-	2	-	43	-	34	-	43	-							17	-	83
	t5017	8	-	34	-	2	-	43	-	34	-	43	-	43	-	16	-	2	-	17	-	83
	t4981	8	-	16	-	2	-	43	-	17	-							34				
	t5018	8	-	16	-	2	-	43	-	17	-	173	-	34	-	17	-	34				
8	t4168	8	-	23	-	21	-	17	-	13	-	13	-	13	-	24	-	23				
	t5010	8	-	23	-	12	-	17	-	13	-	13	-	13	-	24	-	23				
9	t4167	7	-	23	-	17	-	22	-	249	-	12	-	117	-	24	-	25	-	17		
	t4168	8	-	23	-	21	-	17	-	13	-	13	-	13	-	24	-	23				
	t5015	15	-	12	-	16	-	16	-	25	-	25	-	34	-	24	-	17	-	17	-	17
	t5137	4	-	16	-	13																
10	t4168	8	-	23	-	21	-	17	-	13	-	13	-	13	-	24	-	23				
11	t189	7	-	23	-	12	-	21	-	17	-	34										

**Figure 2:**
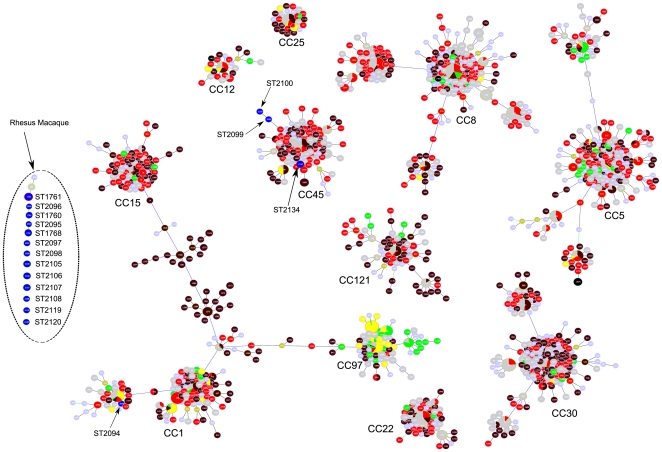
Partial population snapshot of *S. aureus*. This snapshot was created by goeBURST v1.2 software using the dataset downloaded from http://saureus.mlst.net/ that included 2010 STs representing 3887 isolates. A subset of this dataset (1304 STs, representing 2875 isolates) is presentend in this figure including all major Clonal Complexes (CCs) supplemented with all rhesus macaque ST as far as they were not part of the major CCs. The area of each circle in the goeBURST diagram corresponds to the relative abundance of the STs in the input data. The names of major CCs have been indicated. ST colors refer to the source *S. aureus* was isolated from. STs representing macaque isolates are indicated in blue and by their ST number; brown colors correspond with community-acquired human isolates; red colors correspond with hospital-acquired human isolates; green colors correspond with animal isolates; yellow colors correspond with *S. aureus* isolates from food; grey, light green, and light blue colors correspond with isolates with unknown source.

For one *S. aureus* isolate of each *spa* type, we performed MLST and compared STs found in macaque strains with all STs available in the *S. aureus* MLST database at the moment of interrogation (Figure 2). Seventeen different STs were found, which were all not present in the *S. aureus* MLST database at the time of interrogation. ST2099, ST2100 and ST2134 were part of CC45, which mostly contained STs found in human *S. aureus* isolates. ST2099 and ST2100 were SLVs. Isolates with these 3 STs were found in different PFGE clusters. ST2094 had relations with STs in CC1, which contained a lot of STs found in human isolates as well. The PFGE pattern of this *S. aureus* isolate differed from all other macaque isolates, as this isolate was found in PFGE cluster 11, in which this isolate was the only one from macaque origin. All other STs found in the macaque isolates (ST1760, ST1761, ST1768, ST2095, ST2096, ST2097, ST2098, ST2105, ST2106, ST2107, ST2108, ST2119, ST2120) were not part of the larger CCs, but were present as singletons. ST1760 and ST2096 as well as ST1768 and ST2095 were SLVs, and were both found in PFGE clusters 7 and 6, respectively, clusters with mainly isolates from macaques. Isolates with STs present as a singleton were distributed among PFGE clusters 4 to 9, which were clusters with mainly isolates from rhesus macaques.

Next, we determined the genomic presence of *mecA*, 19 superantigen genes, and *eta* and *etd* by PCR. None of the 108 selected isolates harboured the *mecA* gene, which indicates that none of these isolates were MRSA. Thirty-two isolates that were representative for each *spa* type were selected for testing presence of superantigen genes and *eta* and *etd* (Table 3). These genes were absent in 66% (21/32) of these isolates. The *etd* gene was detected in 9% of the isolates (3/32), 16% of the isolates were *sei*-positive (5/32), 19% were positive for *sem*, *sen*, and *seo* (6/32), and 25% were positive for *seg* (8/32). Genes encoding SEA to SEE, SEH, SEJ to SEL, SEP to SER, SEU, TSST-1 and ETA were absent in all 32 isolates. Only 6 isolates harboured a complete or incomplete *egc* locus. Four of these were strains from PFGE clusters comprising only macaque isolates, while 2 isolates belong to clusters including both macaque and human isolates.

**Table 3:** Presence of genes encoding superantigens, exfoliative toxins, and *agr* types in 32 representative *S. aureus* strains isolated from rhesus macaques.

	Gene^a^												
Isolate no.	*seg*	*sei*	*sem*	*sen*	*seo*	*etd*	*agr-1*	*agr-2*	*agr-3*	*agr-4*	Gene profile	PFGE cluster	Origin isolates in PFGE cluster^b^
08-1178	−	−	−	−	−	−	+	−	−	−	1	5	RM
08-1192	−	−	−	−	−	−	+	−	−	−	1	4	RM
08-1276	−	−	−	−	−	−	+	−	−	−	1	7	RM
08-1494	−	−	−	−	−	−	+	−	−	−	1	7	RM
08-1495	−	−	−	−	−	−	+	−	−	−	1	7	RM
08-1185	+	+	+	+	+	−	+	−	−	−	2	8	RM
08-1415	+	+	+	+	+	−	+	−	−	−	2	8	RM
08-1197	+	−	−	−	−	−	−	−	−	+	3	6	RM+HV
08-1255	+	−	−	−	−	−	−	−	−	−	4	11	RM+HV
08-1299	−	−	−	−	−	−	−	−	−	+	5	5	RM
08-1514	−	−	−	−	−	−	−	−	−	+	5	5	RM
08-1332	−	−	−	−	−	−	−	+	−	−	6	4	RM
08-1342	−	−	−	−	−	−	−	−	+	−	7	6	RM+HV
08-1404	−	−	−	−	−	−	−	−	−	−	8	5	RM
08-1412	−	−	−	−	−	−	−	−	−	−	8	6	RM+HV
08-1423	−	−	−	−	−	−	−	−	−	−	8	7	RM
08-1430	−	−	−	−	−	−	−	−	−	−	8	7	RM
08-1432	−	−	−	−	−	−	−	−	−	−	8	7	RM
08-1487	−	−	−	−	−	−	−	−	−	−	8	6	RM+HV
08-1504	−	−	−	−	−	−	−	−	−	−	8	7	RM
08-1507	−	−	−	−	−	−	−	−	−	−	8	7	RM
08-1528	−	−	−	−	−	−	−	−	−	−	8	7	RM
08-1737	−	−	−	−	−	−	−	−	−	−	8	2	RM+HV
08-1749	−	−	−	−	−	−	−	−	−	−	8	7	RM
08-1756	−	−	−	−	−	−	−	−	−	−	8	7	RM
08-1419	−	−	−	−	−	+	−	−	+	−	9	7	RM
08-1420	−	−	−	−	−	+	−	−	+	−	9	7	RM
08-1486	+	−	+	+	+	−	−	−	−	−	10	7	RM
08-1583	+	+	+	+	+	−	−	−	−	−	11	6	RM+HV
08-1602	+	+	+	+	+	−	−	−	+	−	12	9	RM+HV
08-1765	+	+	+	+	+	−	−	−	+	−	12	7	RM
08-1630	−	−	−	−	−	+	−	−	−	−	13	2	RM+HV
No. of positive isolates (%)	8 (25)	5 (16)	6 (19)	6 (19)	6 (19)	3 (9)	7 (22)	1 (3)	5 (16)	3 (9)			

a All isolates were negative for *sea*, *seb*, *sec*, *sed*, *see*, *seh*, *sej*, *sek*, *sel*, *sep*, *seq*, *ser*, *seu*, *tst*, and *eta*.

b Isolates belong to typically rhesus macaque (RM) PFGE clusters or to mixed clusters including both macaque and healthy human volunteer (HV) strains.

These 32 isolates were also *agr* genotyped (Table 3). The most common *agr* type was type I (22%), followed by *agr* type III (16%), type IV (9%), and type II (3%). Sixteen isolates (50%) did not belong to any of the 4 known *agr* types, which is intriguing. Isolates with *agr* type I or type II belonged to PFGE clusters comprising only macaque isolates, while isolates with other *agr* types belonged to clusters including both macaque and human isolates. Overall, 13 combinations of *agr* type and toxin genes were found. Two of the *agr* type I isolates, two of the *agr* type III isolates and two of the unknown *agr* type isolates harboured a complete or incomplete *egc* locus. Five *agr* type I isolates, the single *agr* type II isolate, one *agr* type III isolate, two *agr* type IV isolates, and 12 isolates with an unknown *agr* type harboured none of the superantigen and exfoliative toxin genes. *S. aureus* isolates with the same *agr* type and toxin genes were distributed among the different PFGE clusters.

### *S. aureus* nasal carriage in rhesus macaques

Forty-eight (4 groups of 12) macaques were screened three times for nasal carriage of *S. aureus* over a 5 month period. The four groups of twelve macaques were housed in separate rooms. For 9 macaques (18.8%), all nasal culture results were negative. Eighteen macaques (37.5%) had one positive culture, 13 (27.1%) had two positive cultures, and for 8 macaques (16.7%) *S. aureus* was found in all three cultures.

**Figure 3:**
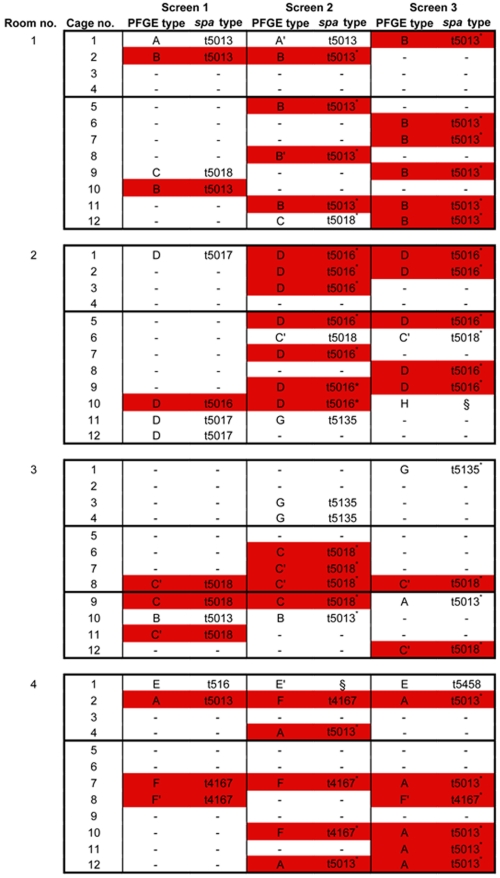
Persistence of *S. aureus* nasal carriage in rhesus macaques. Macaques were duo-housed. Physical contact to the macaques in the neighbouring cage was possible. Each block indicates an animal room, horizontal lines in blocks indicate which macaques were not able to have physical contact. Red color indicates the “epidemic” strain in each room. – indicates a *S. aureus* negative nasal culture. * indicates that the isolate was not *spa* genotyped itself, *spa* type was derived from typed isolates with identical PFGE pattern. § indicates that the isolate was not *spa* genotyped, and this could not be derived from typed isolates with identical PFGE pattern. *S. aureus* strains were frequently exchanged between macaques in the same room.

Isolates from these 48 duo-housed macaques were also typed by PFGE, and *spa* types were determined for representative isolates from each PFGE cluster (Figure 3). In macaques with more than one *S. aureus* positive nasal culture, a strain replacement occurred in half of the animals.

It seemed that there was one most prevalent “epidemic” strain in each animal room. This strain spread from one or a couple of macaques to most others. This phenomenon was clear for rooms 1 (PFGE type B, *spa* type t5013), 2 (PFGE type D, *spa* type t5016) and 3 (PFGE type C′, *spa* type t5018), while for room 4, the epidemic strain was less clear (PFGE type A, *spa* type t5013, or PFGE type F, *spa* type t4167). In some cases, a macaque did not acquire the epidemic strain, e.g. the macaque in animal room 2, cage 6 had two nasal cultures positive with a *S. aureus* isolate with PFGE type C′, while the epidemic isolate had PFGE type D.

### Anti-staphylococcal antibodies in rhesus macaques

**Figure 4:**
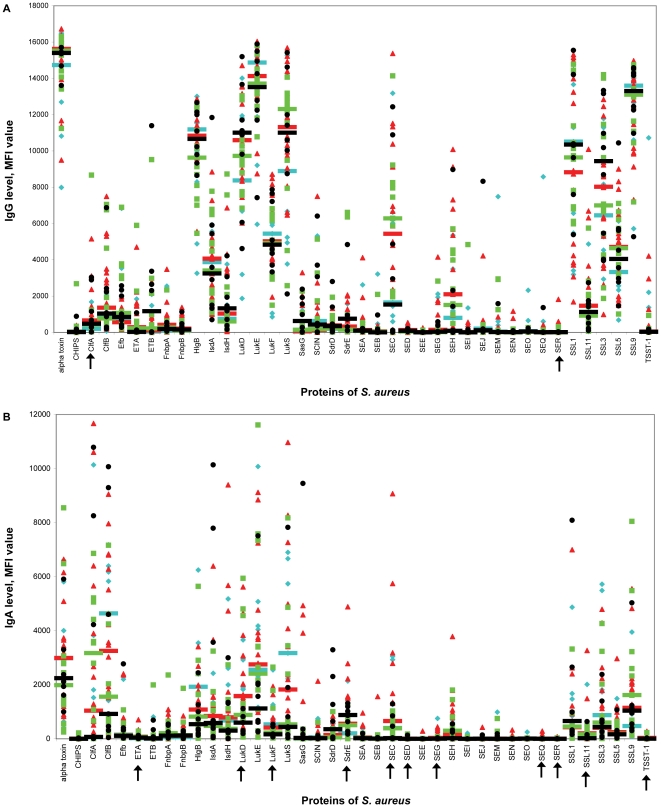
Relation between *S. aureus* nasal colonization and level of anti-staphylococcal IgG (A) and IgA (B). Antibody levels are reflected by Median fluorescence intensity (MFI) value. Each symbol represents a single rhesus macaque. Blue diamonds represent macaques without *S. aureus* positive culture, red triangles represent macaques with one positive culture, green squares represent macaques with two positive cultures, and black circles represent macaques with three positive cultures. Median values are indicated by horizontal lines. Arrows indicate statistically significant differences in median values (Mann-Whitney *U* test). More *S. aureus* positive cultures were not related to high – or low – anti-staphylococcal antibody levels. CHIPS, chemotaxis inhibitory protein of *S. aureus*; Clf, clumping factor; Efb, extracellular fibrinogen-binding protein; ET, exfoliative toxin; Fnbp, fibronectin-binding protein; HlgB, γ hemolysin B; Isd, iron-responsive surface determinant; Luk, leukocidin; SasG, *S. aureus* surface protein G; SCIN, staphylococcal complement inhibitor; Sdr, serine-aspartate dipeptide repeat protein; SE, staphylococcal enterotoxin; SSL, staphylococcal superantigen-like protein; TSST-1, toxic shock syndrome toxin 1.

Serum samples from 47 of the 48 macaques that were screened thee times for nasal carriage were analyzed for anti-staphylococcal antibodies directed against 40 different *S. aureus* proteins. The MFI values reflecting the serum IgG and IgA levels for each macaque and each antibody isotype are shown in Figure 4. An extensive diversity in individual antibody responses was observed, similar to the findings in humans. Individual macaques had high antibody levels against a number of antigens, while anti-staphylococcal antibody levels against other antigens were low.

For most antigens, there was no apparent quantitative difference in antibody level between macaques with 0, 1, 2, or 3 positive cultures. However, the median serum levels of IgG directed against ClfA were significantly higher in rhesus macaques with one positive culture than in those without positive culture (MFI value, 657 vs. 191; *P*<0.05). Additionally, median IgA levels were higher in macaques without a positive culture than in those with three positive cultures for LukF (532 vs. 167; *P*<0.05). In macaques with three positive cultures, median IgA levels were higher than in macaques without positive culture for SdrE (881 vs. 200; *P*<0.05). In macaques with one positive culture, median IgA levels were higher than in macaques with three positive cultures for LukD (1580 vs. 599; *P*<0.005), SEC (658 vs. 23; *P*<0.05), and SSL11 (204 vs 22; *P*<0.05). In general, a larger number of positive cultures was not related to elevated or decreased anti-staphylococcal antibody levels. Other comparisons were also made (non-carriers *vs.* intermittent *vs.* persistent carriers; carriers of the most prominent isolate in the animal room *vs.* carriers of another isolate). Again, no association was found between nasal carrier status and anti-staphylococcal antibody levels in serum (data not shown).

**Figure 5:**
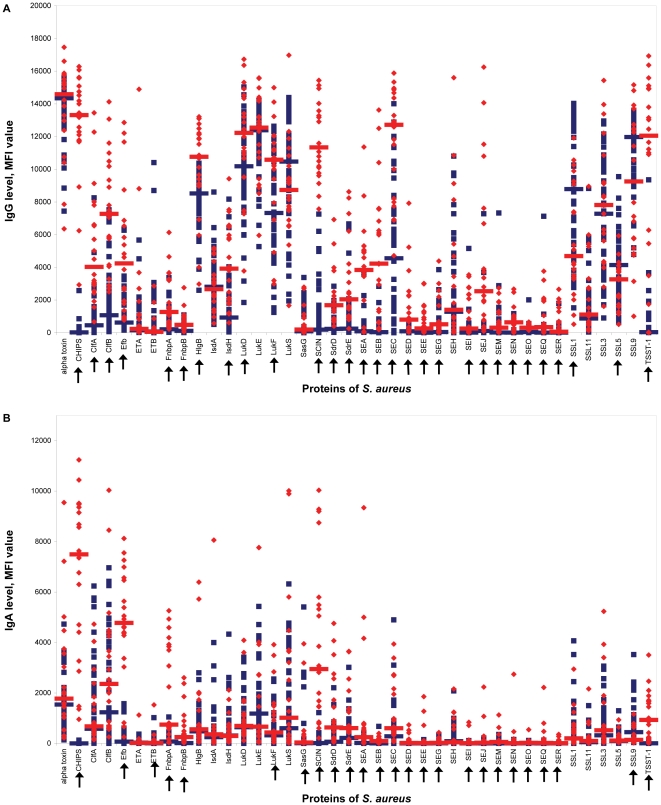
Level of anti-staphylococcal IgG (A) and IgA (B) in rhesus macaques and human volunteers. Antibody levels are reflected by Median fluorescence intensity (MFI) value. Each symbol represents a single rhesus macaque or human. Blue squares represent healthy rhesus macaques and red diamonds represent healthy human volunteers. Median values are indicated by horizontal lines. Arrows indicate statistically significant differences in median values (Mann-Whitney *U* test). Anti-staphylcoccal IgG and IgA levels were significantly different between rhesus macaques and humans for 29 and 25 out of 40 antigens, respectively. CHIPS, chemotaxis inhibitory protein of *S. aureus*; Clf, clumping factor; Efb, extracellular fibrinogen-binding protein; ET, exfoliative toxin; Fnbp, fibronectin-binding protein; HlgB, γ hemolysin B; Isd, iron-responsive surface determinant; Luk, leukocidin; SasG, *S. aureus* surface protein G; SCIN, staphylococcal complement inhibitor; Sdr, serine-aspartate dipeptide repeat protein; SE, staphylococcal enterotoxin; SSL, staphylococcal superantigen-like protein; TSST-1, toxic shock syndrome toxin 1.

Isolates from rhesus macaques differ from those from humans in PFGE type, *spa* type, *agr* type, and prevalence of superantigen genes. To determine whether these different *S. aureus* isolates induce different humoral antibody responses, anti-staphylococcal antibody levels in sera of the 47 rhesus macaques were compared to those in sera of 20 healthy human volunteers (Figure 5). Significant differences in anti-staphylococcal IgG and IgA levels between rhesus macaques and healthy volunteers were observed for 29 and 25 out of 40 antigens, respectively (Table 4). In general, the median antibody serum levels in humans were higher than those in macaques. However, the median serum IgG level directed against SSL1 and SSL5 was significantly higher in macaques than in humans. The median serum IgA level directed against SSL9 was significantly higher in macaques than in humans.

**Table 4:** Median fluorescence intensity (MFI) values reflecting antigen-specific IgG and IgA levels in rhesus macaques (RM) and healthy human volunteers (HV).

	MFI value, median (range)				MFI value, median (range)		
*S. aureus* protein (antibody isotype)	RM	HV	*P* a	*S. aureus* protein (antibody isotype)	RM	HV	*P* a
alpha toxin (IgG)	14337 (7438–15574)	14587 (6355–17465)	0.285	SEA (IgG)	88 (17–3565)	3839 (29–11365)	<0.001
alpha toxin (IgA)	1540 (139–4729	1775 (215–9549)	0.154	SEA (IgA)	16 (1–449)	247 (5–9345)	<0.001
CHIPS (IgG)	20 (0–2580)	13310 (2933–16281)	<0.001	SEB (IgG)	34 (0–2836)	4223 (190–13622)	<0.001
CHIPS (IgA)	1 (0–130)	7494 (957–11238)	<0.001	SEB (IgA)	2 (0–673)	90 (2–398)	<0.001
ClfA (IgG)	454 (16–8250)	4029 (670–13449)	<0.001	SEC (IgG)	4551 (1–14006)	12720 (4713–15884)	<0.001
ClfA (IgA)	580 (4–6237)	673 (145–4730)	0.373	SEC (IgA)	280 (0–4896)	600 (63–3943)	0.004
ClfB (IgG)	1066 (65–7518)	7272 (2499–14127)	<0.001	SED (IgG)	84 (3–533)	800 (33–7924)	<0.001
ClfB (IgA)	1232 (0–6962)	2357 (255–10038)	0.125	SED (IgA)	1 (0–85)	19 (0–736)	<0.001
Efb (IgG)	622 (162–6325)	4238 (770–12855)	<0.001	SEE (IgG)	3 (0–229)	258 (7–3017)	<0.001
Efb (IgA)	69 (0–1575)	4775 (257–8125)	<0.001	SEE (IgA)	0 (0–151)	12 (0–1856)	<0.001
ETA (IgG)	156 (10–5670)	234 (16–14891)	0.217	SEG (IgG)	37 (0–3861)	517 (5–4385)	0.002
ETA (IgA)	30 (1–366)	19 (6–1127)	0.742	SEG (IgA)	5 (0–353)	22 (0–432)	0.035
ETB (IgG)	72 (0–10405)	55 (1–2855)	0.515	SEH (IgG)	1282 (0–10804)	1400 (9–15602)	0.468
ETB (IgA)	3 (0–1023)	20 (1–1529)	0.026	SEH (IgA)	88 (0–2083)	44 (0–2158)	0.821
FnbpA (IgG)	208 (20–3374)	1269 (222–6132)	<0.001	SEI (IgG)	4 (0–5154)	257 (0–3544)	<0.001
FnbpA (IgA)	73 (1–1047)	744 (26–5261)	<0.001	SEI (IgA)	0 (0–111)	8 (0–840)	<0.001
FnbpB (IgG)	111 (9–1089)	478 (54–2758)	<0.001	SEJ (IgG)	105 (5–7274)	2538 (95–16246)	<0.001
FnbpB (IgA)	15 (2–253)	253 (19–2603)	<0.001	SEJ (IgA)	1 (0–217)	25 (0–2236)	<0.001
HlgB (IgG)	8519 (2595–10365)	10761 (2979–13212)	0.001	SEM (IgG)	12 (0–7325)	307 (8–2738)	<0.001
HlgB (IgA)	474 (10–2799)	556 (44–6394)	0.233	SEM (IgA)	1 (0–354)	56 (0–1128)	<0.001
IsdA (IgG)	2811 (508–8602)	2665 (576–5183)	0.732	SEN (IgG)	7 (0–1166)	631 (0–2672)	<0.001
IsdA (IgA)	248 (13–3997)	357 (9–8058)	0.940	SEN (IgA)	0 (0–155)	7 (0–2743)	<0.001
IsdH (IgG)	929 (44–8170)	3921 (605–9420)	<0.001	SEO (IgG)	40 (7–2009)	306 (147–653)	<0.001
IsdH (IgA)	311 (3–4328)	294 (43–2117)	0.661	SEO (IgA)	3 (0–117)	20 (4–544)	<0.001
LukD (IgG)	1082 (1852–15031)	12226 (3781–16731)	0.011	SEQ (IgG)	5 (0–7125)	341 (0–3763)	<0.001
LukD (IgA)	641 (0–3418)	694 (106–4088)	0.753	SEQ (IgA)	0 (0–132)	19 (0–2219)	<0.001
LukE (IgG)	12386 (5270–14191)	12541 (5955–15590)	0.732	SER (IgG)	3 (0–1797)	63 (0–2654)	<0.001
LukE (IgA)	1181 (28–5431)	664 (38–7766)	0.524	SER (IgA)	0 (0–40)	9 (0–357)	0.001
LukF (IgG)	7321 (1240–12631)	10579 (2984–14996)	0.007	SSL1 (IgG)	8783 (1520–14022)	4683 (518–11914)	0.005
LukF (IgA)	321 (0–2554)	433 (38–3916)	0.041	SSL1 (IgA)	198 (0–4066)	195 (6–2947)	0.956
LukS (IgG)	10470 (1939–14385)	8734 (1662–16976)	0.135	SSL11 (IgG)	867 (43–8824)	1107 (122–8955)	0.311
LukS (IgA)	602 (0–6321)	1015 (13–10014)	0.524	SSL11 (IgA)	46 (3–1495)	96 (8–2162)	0.215
SasG (IgG)	89 (3–3376)	197 (3–2791)	0.093	SSL3 (IgG)	7280 (898–12949)	7813 (1618–15432)	0.403
SasG (IgA)	8 (0–5408)	33 (4–3950)	0.009	SSL3 (IgA)	321 (5–3062)	519 (24–5234)	0.244
SCIN (IgG)	187 (5–7259)	11333 (7572–15438)	<0.001	SSL5 (IgG)	4138 (621–9547)	3267 (511–5928)	0.021
SCIN (IgA)	14 (0–1220)	2945 (211–10036)	<0.001	SSL5 (IgA)	97 (6–1293)	115 (27–933)	0.264
SdrD (IgG)	233 (17–2844)	1683 (400–6924)	<0.001	SSL9 (IgG)	11968 (4788–13605)	9255 (1005–15161)	0.150
SdrD (IgA)	64 (3–2205)	634 (48–4758)	<0.001	SSL9 (IgA)	444 (35–3523)	138 (20–1158)	0.001
SdrE (IgG)	250 (28–6655)	2045 (251–8626)	<0.001	TSST-1 (IgG)	32 (4–9350)	12051 (0–16927)	<0.001
SdrE (IgA)	218 (0–3017)	604 (28–3650)	0.015	TSST-1 (IgA)	4 (0–125)	932 (10–3504)	<0.001

a Differences in antigen-specific MFI values between groups were considered to be statistically significant at *P*<0.05 (Mann-Whitney *U* test).

### Association of anti-staphylococcal antibody levels with presence of genes

To study whether the low antibody levels in rhesus macaques were due to absence of the genes encoding the antigens, 10 isolates from the macaques that were screened three times, were selected for determination of the presence of genes encoding antigens against which a low MFI value was found in macaques, while MFI values in humans were high (SCIN, CHIPS, ClfA, ClfB, Efb, TSST-1, FnbpA, and FnbpB). Results are shown in Table 5. None of the isolates from rhesus macaques were *scn*-, *tst*-, or *fnbB*-positive, which explains why antibody levels were at background level. On the other hand, all 10 isolates were *clfB*- and *efb*-positive, and the macaques that carry these isolates had high antibody levels against these antigens. Nine out of 10 *S. aureus* isolates did not possess the gene encoding for CHIPS, which explains why the MFI values were at background level. The macaque from which the *chp*-positive strain was isolated also had MFI values at background level. Eight isolates were *clfA*-positive, with corresponding high MFI values for 6 macaques. Interestingly, the two macaques from which the *clfA*-negative strains were isolated, still had high MFI values. Nine isolates possessed the gene encoding FnbpA, which corresponds with MFI values just above background level (range 22–616). However, the macaque with the *fnbA*-negative *S. aureus* strain had MFI values above background level as well. These differences are probably due to a previous exposure to an other *S. aureus* strain.

**Table 5:** Association of anti-staphylococcal antibody levels with presence of genes in 10 *S. aureus* strains isolated from rhesus macaques.

	SCIN			CHIPS			ClfA			ClfB		
Isolate no.	Gene	MFI value, IgG	MFI value, IgA	Gene	MFI value, IgG	MFI value, IgA	Gene	MFI value, IgG	MFI value, IgA	Gene	MFI value, IgG	MFI value, IgA
1	−	356	2	−	6	0	−	664	82	+	291	45
2	−	6404	77	−	23	1	+	2893	8252	+	6871	10067
3	−	5	3	−	15	1	+	3033	10785	+	997	4604
4	−	305	2	−	8	0	+	142	660	+	816	238
5	−	759	44	−	65	0	+	275	5213	+	1382	805
6	−	3702	26	+	173	3	−	282	4223	+	2423	9292
7	−	3067	127	−	30	2	+	26	7	+	866	462
8	−	492	11	−	886	0	+	27	37	+	2232	310
9	−	155	9	−	338	11	+	664	3549	+	462	549
10	−	27	76	−	8	27	+	17	1574	+	192	1574

	Efb			TSST-1			FnbpA			FnbpB		
Isolate no.	Gene	MFI value, IgG	MFI value, IgA	Gene	MFI value, IgG	MFI value, IgA	Gene	MFI value, IgG	MFI value, IgA	Gene	MFI value, IgG	MFI value, IgA
1	+	1011	107	−	14	2	+	60	36	−	12	23
2	+	2332	2773	−	58	27	+	545	254	−	477	169
3	+	2564	402	−	180	9	+	181	100	−	1132	255
4	+	1156	101	−	10	0	+	157	56	−	78	86
5	+	373	48	−	43	16	+	165	616	−	63	223
6	+	279	321	−	37	0	+	192	544	−	161	300
7	+	416	25	−	30	22	+	131	22	−	324	13
8	+	679	61	−	37	1	+	508	134	−	149	74
9	+	3777	1069	−	197	5	+	74	51	−	24	35
10	+	2003	1061	−	8	4	−	356	502	−	26	263

## Discussion

Nasal carriage of *S. aureus* plays a key role in the epidemiology and pathogenesis of staphylococcal infections [27], [28]. Eradication of *S. aureus* from the nose has proven to be effective in the reduction of staphylococcal infections [29]–[32]. This indicates that the anterior nasal region is a primary ecological reservoir of *S. aureus* [33], [34], although throat and perineum are important reservoirs as well [35]–[37]. However, nasal re-colonization may occur within weeks to months in those who have successfully been decolonized [38], [39]. In order to develop new strategies in prevention of staphylococcal disease, acquiring additional knowledge about the underlying mechanisms of *S. aureus* nasal carriage is important. Artificial inoculation of human volunteers offers opportunities to study these mechanisms [40]. While such semi-clinical studies in humans remain the most informative, animal models of *S. aureus* colonization sometimes enable a more detailed investigation of the processes involved in pathogenesis by allowing for more risky interventions.

In the present study, we describe for the first time natural nasal *S. aureus* carriage in rhesus macaques. The nasal cavity of these macaques appears to be an important reservoir of *S. aureus*, as is also observed in humans. In a single screening of their noses, 39% of the 731 rhesus macaques had a positive culture, which is comparable to the human situation with ∼20% persistent carriers and ∼30% intermittent carriers [28], [41], [42]. Most of these *S. aureus* isolates were different from human *S. aureus* isolates: rhesus macaque isolates formed separate PFGE clusters, 59% had one of the 17 *spa* types that were not yet described, the previously described *spa* types t189, t516, t729, t786, and t1814 were rare as well (Ridom SpaServer), in most macaque isolates genes encoding superantigens were not present, and half of the isolates could not be *agr* typed. Moreover, in the majority of macaque isolates new STs were found which were not related to described STs. Three isolates were part of CC45, and one was part of CC1. These macaque isolates were therefore more comparable to human isolates than the other ones. Differences between macaque and human *S. aureus* isolates are underlined by major differences in both anti-staphylococcal antibody levels and gene content of the *S. aureus* isolates between these two species. This indicates that new host-specific lineages of *S. aureus* have now been found in rhesus macaques.

In contrast to the macaque *S. aureus* isolates, most human strains harbour at least one gene encoding superantigens, on average 5 or 6 genes, among which those encoding the *egc* superantigens SEG, SEI, SEM, SEN, and SEO are most prevalent [23], [43], [44]. These genes were also most prevalent in the macaque isolates. The relatively low number of isolates containing superantigen genes could also be due to the fact that the primers used in this study were designed for *S. aureus* strains isolated from humans. Therefore, it can not be concluded that most macaque *S. aureus* isolates do not carry superantigen genes at all, but that, at least, if they are present, these genes differ from those found in human *S. aureus* isolates.

In addition, half of the macaque isolates could not be typed with the current *agr* multiplex PCR system. Of those that could be typed, most belonged to *agr* types I or III, which is in contrast to human *S. aureus* isolates. These *agr* types are also the largest groups (*agr* type I 35% and type III 38%), but 25% of the isolates contains *agr* type II [45]. In contrast, *agr* type IV is hardly found among human isolates (2%), while 19% of the typeable macaque isolates harboured this type. The *agr* untypeable *S. aureus* isolates probably have a deletion of the *agr* locus or extensive sequence variation at the primer sites and could therefore not be typed. This further emphasizes that *S. aureus* isolates in rhesus macaques differ from those in humans.

Next, we studied the persistence of nasal *S. aureus* carriage in 48 rhesus macaques. PFGE and *spa* typing of these isolates, as well as the absence of certain antigen genes while serum anti-staphylococcal antibody levels against these antigens were high, suggest that persistent nasal *S. aureus* carriage as observed in humans is not present among these rhesus macaques. Human persistent carriers are usually colonized by the same *S. aureus* strain over a long time period [46], while half of the studied rhesus macaques carried different *S. aureus* isolates, even over a 5 month period. Frequent transmission of *S. aureus* isolates rather than persistent nasal carriage was suggested by the presence of one or two epidemic strains in each animal room.

The absence of persistent nasal carriage was underlined by the absence of an association between nasal carrier status and anti-staphylococcal antibody levels in serum of rhesus macaques, as was observed in humans. Differences in IgG levels between human persistent carriers and non-carriers were reported for alpha hemolysin, major autolysin, IsdA and IsdH, immunodominant secretory antigen A (IsaA), major histocompatibility complex class II analogue protein w (Map-w), ClfB, TSST-1, and SEA. IgA levels were different between persistent carriers and non-carriers for TSST-1, SEA, ClfA, and ClfB [14], [47], [48].

In addition, the differences in anti-staphylococcal antibody levels in rhesus macaques and humans, independent of nasal carrier status, again underlined the differences between *S. aureus* isolates in humans and those in macaques. The major immunogenic proteins in human *S. aureus* strains do not seem to elicit a humoral immune response in rhesus macaques.

In conclusion, the present study demonstrates that rhesus macaques are natural hosts of *S. aureus*, and that *S. aureus* isolates from rhesus macaques versus those from humans differ in many aspects. Therefore, rhesus macaques are not suitable for studying *S. aureus* persistent carriage as is seen in humans. However, the nasal cavity is an important reservoir in both species, which implies that the rhesus macaque provides an interesting model for studying short term nasal colonization, and in particular bacterial factors involved in adherence. Furthermore, rhesus macaque provide an autologous system in which transmission of *S. aureus* strains between individuals can be studied, and are therefore a useful model for studying infection prevention. Whether data from these studies can be directly extrapolated to humans is unclear. However, possibilities for extrapolation from rhesus macaque to human appear reasonable because of their close evolutionary relatedness.
