# Correction: Rhesus Macaques (*Macaca mulatta*) Are Natural Hosts of Specific *Staphylococcus aureus* Lineages

**DOI:** 10.1371/annotation/bc0581bd-5ef5-4116-b3b6-6fc6c5eb197c

**Published:** 2012-08-28

**Authors:** Sanne van den Berg, Willem J. B. van Wamel, Susan V. Snijders, Boudewijn Ouwerling, Corné P. de Vogel, Hélène A. Boelens, Rob J. L. Willems, Xander W. Huijsdens, Frank A. W. Verreck, Ivanela Kondova, Peter J. Heidt, Henri A. Verbrugh, Alex van Belkum

The errors in the "Characterization of S. aureus isolates in rhesus macaques" section of Results are:

- Sixteen (instead of seventeen) different STs were found, of which 15 were not present in the S. aureus MLST database at the time of interrogation. ST188 was already described in human S. aureus isolates.

- ST188 (instead of ST2094) had relations with STs in CC1, which contained a lot of STs found in human isolates as well.

- ST2097 was part of CC398. All other STs found in the macaque isolates (ST1760, ST1761, ST1768, ST2096, ST2098, ST2105, ST2106, ST2107, ST2108, ST2119, ST2120) were not part of the larger CCs, but were present as singletons.

The errors in Figure 2 are:

- ST2094 should be ST188. The clustering does not alter.

- ST2095 disappears from this Figure.

- ST2097 is not a singleton, but clusters in CC398. 

